# Reduced Graphene Oxide-Supported SrV_4_O_9_ Microflowers with Enhanced Electrochemical Performance for Sodium-Ion Batteries

**DOI:** 10.3390/molecules29112704

**Published:** 2024-06-06

**Authors:** Guangming Li, Yifan Li, Yi Zhang, Shuguo Lei, Jiwei Hou, Huiling Lu, Baizeng Fang

**Affiliations:** 1 CNG Wind Energy Co., Ltd., Beijing 100160, China; 2 School of Physical and Mathematical Science, Nanjing Tech University, Nanjing 211816, China; 3 School of Chemistry and Chemical Engineering, Jiangsu University, Zhenjiang 212013, China; lhl@ujs.edu.cn; 4 School of Chemical Engineering and Energy Technology, Dongguan University of Technology, Dongguan 523808, China

**Keywords:** SrV_4_O_9_, rGO, sodium-ion batteries, anode, electrochemical performance

## Abstract

Sodium-ion batteries (SIBs) have received considerable attention in recent years. Anode material is one of the key factors that determine SIBs’ electrochemical performance. Current commercial hard carbon anode shows poor rate performance, which greatly limits applications of SIBs. In this study, a novel vanadium-based material, SrV_4_O_9_, was proposed as an anode for SIBs, and its Na^+^ storage properties were studied for the first time. To enhance the electrical conductivity of SrV_4_O_9_ material, a microflower structure was designed and reduced graphene oxide (rGO) was introduced as a host to support SrV_4_O_9_ microflowers. The microflower structure effectively reduced electron diffusion distance, thus enhancing the electrical conductivity of the SrV_4_O_9_ material. The rGO showed excellent flexibility and electrical conductivity, which effectively improved the cycling life and rate performance of the SrV_4_O_9_ composite material. As a result, the SrV_4_O_9_@rGO composite showed excellent electrochemical performance (a stable capacity of 273.4 mAh g^−1^ after 200 cycles at 0.2 A g^−1^ and a high capacity of 120.4 mAh g^−1^ at 10.0 A g^−1^), indicating that SrV_4_O_9_@rGO composite can be an ideal anode material for SIBs.

## 1. Introduction

The widespread use of fossil fuels has created several energy and environmental issues that threaten the sustainable development of human society. It is important to develop renewable energy to overcome environmental problems. However, renewable energy—solar, tidal, and wind—is generally unstable, and requires the support of energy storage systems. Among various energy storage systems, lithium-ion batteries (LIBs) are widely used in consumer electronics and power tools due to their high energy density, environmental friendliness, and no memory effect [1,2,3,4,5,6,7,8]. However, due to required expensive rare metals, such as lithium and cobalt, LIBs cannot well meet the demand for widespread energy storage.

In recent years, sodium-ion batteries (SIBs) have received considerable attention from international researchers due to their many advantages: (1) sodium resources are abundant and widely distributed [9,10,11], (2) SIBs have similar working mechanisms to LIBs so they can be manufactured by the same technologies as LIBs [12], (3) to achieve the same ionic conductivity, the concentration of sodium salts in electrolytes is lower than that of lithium salts, resulting in lower cost, (4) aluminum foil can be used as the current collector for the anode in SIBs because Na^+^ does not react with aluminum, further reducing the cost, and (5) SIBs have high safety performance due to higher internal resistance compared to LIBs. Therefore, SIBs are considered as an alternative to LIBs in some areas.

The anode material is one of the key factors determining the electrochemical performance of batteries [13,14,15,16,17,18]. The main anode materials for SIBs include carbon materials, alloy materials, transition metal oxides, and organic compounds. Among them, hard carbons are the most cost effective, making them promising for industrial application. However, the rate performance of hard carbons is poor, which limits their applications [19,20]. Therefore, it is essential to develop anode materials with high Na^+^ diffusion coefficient for SIBs. Vanadium-based materials have been widely studied as anode materials for SIBs [21,22] due to several advantages. Firstly, they exhibit high capacity due to the multivalent nature of vanadium. Secondly, they have a high Na^+^ diffusion coefficient. Finally, vanadium resources are abundant and environmentally friendly. To date, a novel vanadium-based material (SrV_4_O_9_) has been prepared and has shown excellent Zn^2+^ storage performance [23]. However, research into SrV_4_O_9_ as an anode material for SIBs has not been reported.

In this work, the Na^+^ storage performance of SrV_4_O_9_ material was studied to enhance the electrical conductivity of SrV_4_O_9_ material, and a microflower structure was designed. Reduced graphene oxide (rGO) was then introduced as a host to support SrV_4_O_9_ microflowers. The microflower structure effectively reduced electron diffusion distance, thus enhancing the electrical conductivity of the SrV_4_O_9_ material. In addition, the rGO showed excellent flexibility and electrical conductivity, which can effectively improve the cycling life and rate performance of SrV_4_O_9_ material. As a result, the SrV_4_O_9_@rGO anode exhibited excellent cycling performance and remarkable rate performance.

## 2. Results and Discussion

**Figure 1:**
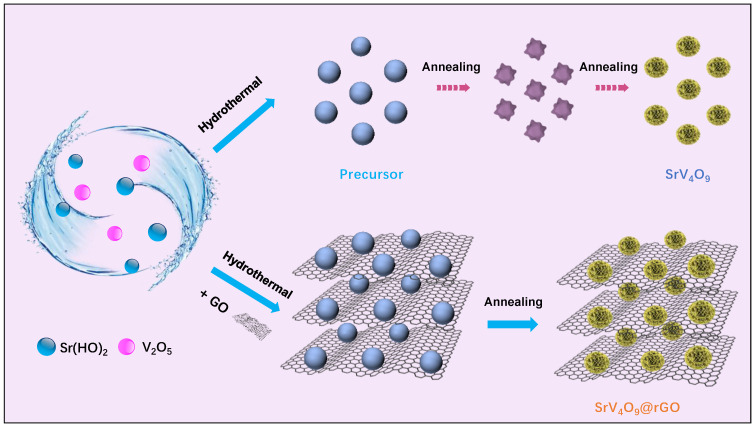
The preparation scheme for SrV_4_O_9_ and the SrV_4_O_9_@rGO composite.

The preparation scheme for SrV_4_O_9_@rGO is shown in Figure 1. The SrV_4_O_9_@rGO shows a microflower structure. The mechanism of formation of the SrV_4_O_9_ flower-like structure involved a combination of factors, including precursor and growth conditions. First, Sr(OH)_2_∙8H_2_O and V_2_O_5_ were dissolved in a solvent and heated at 200 °C for 48 h to form a precursor cluster. The precursor was then heated to 450 °C under argon and held for 5 h. During this process, many branches were formed on the cluster and then grew to eventually form hierarchical structures with intricate morphologies resembling flowers.

**Figure 2:**
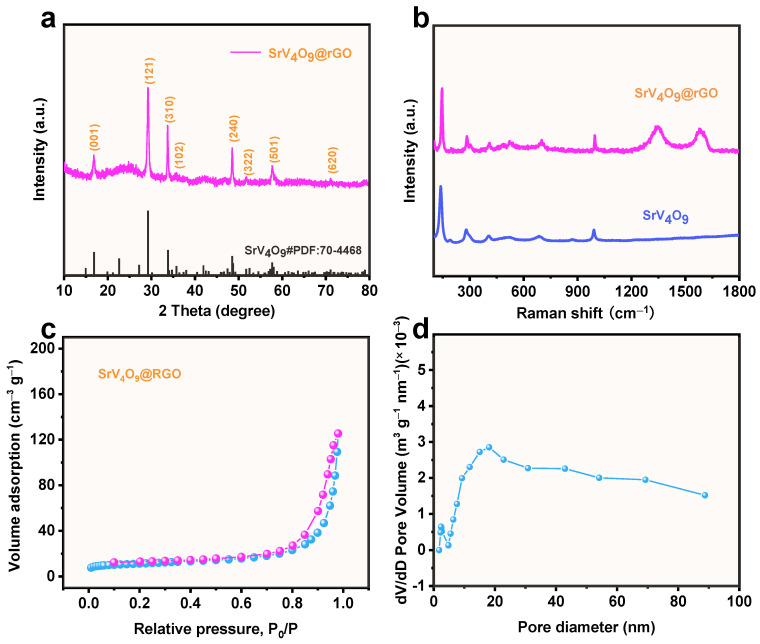
(**a**) XRD pattern of the SrV_4_O_9_@rGO composite. (**b**) Raman spectra of SrV_4_O_9_ and SrV_4_O_9_@rGO. (**c**) N_2_ adsorption–desorption isotherms of SrV_4_O_9_@rGO. (**d**) Pore size distribution of SrV_4_O_9_@rGO.

The structure of the SrV_4_O_9_@rGO composite was investigated by XRD. As shown in Figure 2a, peaks at 16.8°, 29.2°, 33.8°, 35.8°, 48.7°, 52.5°, 57.7°, and 71.1° can be assigned to the (001), (121), (310), (102), (240), (322), (501), and (620) planes of SrV_4_O_9_ (JCPDS No. 70-4468) [23], respectively. Note that rGO diffraction peaks were observed at 26.8° and 42.4°. However, the intensity of the rGO diffraction peaks was weak due to the dense coating of SrV_4_O_9_ on the rGO. To probe the structure of the SrV_4_O_9_@rGO composite, Raman spectroscopy experiments were performed. As seen in Figure 2b, peaks at 148, 292, 380, 524, 682, and 992 cm^−1^ can be attributed to SrV_4_O_9_ [23]. For the SrV_4_O_9_@rGO composite, two additional broad peaks at 1348 and 1578 cm^−1^ corresponded to the D-band and G-band of the rGO, respectively [24,25]. The SrV_4_O_9_@rGO composite was further tested by nitrogen adsorption–desorption experiments to characterize its porosity and specific surface area (Figure 2c,d). The SrV_4_O_9_@rGO composite exhibited a Type I isotherm, indicating abundant micro/mesopores in its structure [26,27]. The specific surface area of the SrV_4_O_9_@rGO composite was 41.4 m^2^ g^−1^. Figure 2d reveals that the average pore size of the SrV_4_O_9_@rGO composite was 17.2 nm, further confirming its porous structure.

**Figure 3:**
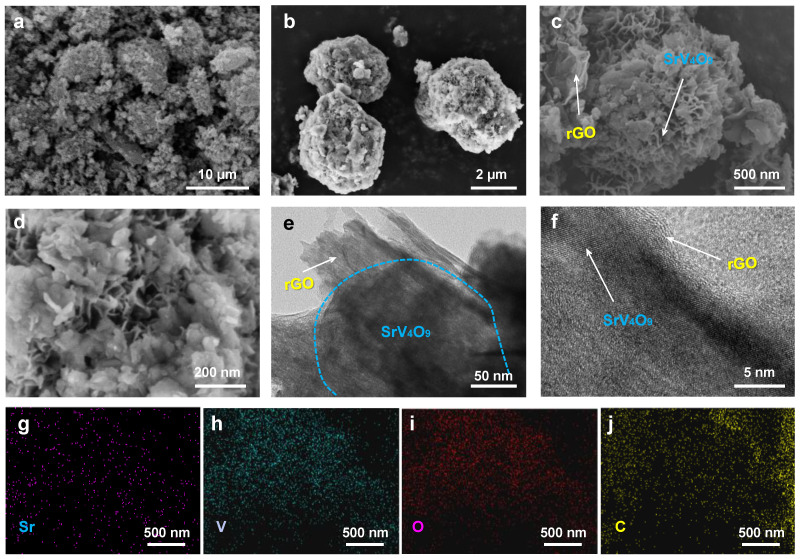
(**a**–**d**) SEM images of the SrV_4_O_9_@rGO composite. (**e**,**f**) TEM images of the SrV_4_O_9_@rGO composite. (**g**–**j**) EDS elemental mapping of Sr, V, O, and C.

To study its morphology, the SrV_4_O_9_@rGO composite was characterized using SEM and TEM. As shown in Figure 3a,b, the SrV_4_O_9_@rGO composite exhibited a three-dimensional porous spherical structure with a diameter of ~6 μm in which flexible rGO coated SrV_4_O_9_ microflowers. Figure 3c shows that SrV_4_O_9_ had a regular micrometer-scale flower-like structure with an average diameter of ~1 µm consisting of porous ultrathin nanosheets with a thickness of ~50 nm (Figure 3d). TEM images (Figure 3e,f) confirmed the three-dimensional porous structure of the SrV_4_O_9_@rGO composite. The porous nanostructure increases the contact area between the active material and the electrolyte and effectively reduces the ion diffusion distance, thus improving ion transport efficiency [28,29,30,31,32]. In addition, the porous nanostructure provides sufficient buffering space for volume expansion during cycling processes, effectively enhancing cycling stability [33,34,35,36,37,38]. As shown in Figure 3g–j, Sr, V, O, and C were uniformly dispersed in the SrV_4_O_9_@rGO composite, indicating that SrV_4_O_9_ was uniformly loaded in rGO.

**Figure 4:**
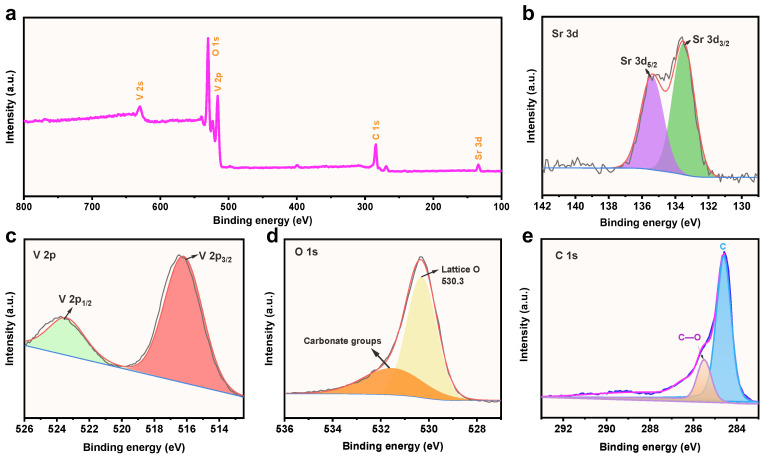
(**a**) The XPS survey spectrum of the SrV_4_O_9_@rGO composite. (**b**) The high-resolution spectrum of Sr 3d. (**c**) The high-resolution spectrum of V 2p. (**d**) The high-resolution spectrum of O 1s. (**e**) The high-resolution spectrum of C 1s.

XPS is a powerful analytical technique for characterizing the chemical composition and oxidation state of materials. The importance of XPS lies in its ability to provide detailed information about the chemical composition of the SrV_4_O_9_@rGO composite at the atomic level. Figure 4a shows the XPS survey spectrum of the SrV_4_O_9_@rGO composite, which shows the photoemission characteristics of Sr, V, O, and C. The high-resolution spectrum of Sr 3d (Figure 4b) can be deconvoluted into Sr^2+^ 3d_5/2_ (135.6 eV) and Sr^2+^ 3d_3/2_ (133.2 eV) [23]. The V 2p high-resolution spectrum (Figure 4c) can be deconvoluted into V^4+^ 2p_1/2_ (523.6 eV) and V^4+^ 2p_3/2_ (516.6 eV) [39,40]. The high-resolution spectrum of O 1s (Figure 4d) exhibits two peaks, at 531.6 eV and 530.4 eV; the peak at 530.4 eV corresponds to the bonding of adsorbed oxygen on the surface of the SrV_4_O_9_@rGO composite with Sr and V, denoted as Sr–O–V [23], while the peak at 531.8 eV was likely due to the presence of H_2_O. As shown in Figure 4e, the high-resolution spectrum of C 1s reveals distinct peaks at 285.6 eV and 284.8 eV, corresponding to C–O and C–C bonds, respectively [41,42]. 

**Figure 5:**
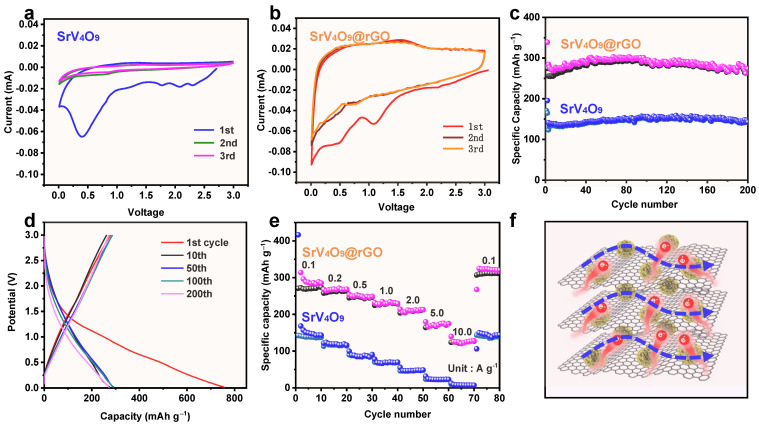
(**a**) CV curves of the SrV_4_O_9_-based electrode for the initial three cycles between 0.01 and 3.0 V at a scan rate of 0.1 mV s^−1^. (**b**) CV curves of SrV_4_O_9_@rGO-based electrodes for the initial three cycles between 0.01 and 3.0 V at a scan rate of 0.1 mV s^−1^. (**c**) Cycling performance of the SrV_4_O_9_-based electrode and the SrV_4_O_9_@rGO-based electrode at 0.2 A g^−1^. (**d**) Voltage profiles of the SrV_4_O_9_@rGO-based electrode at 0.2 A g^−1^. (**e**) Rate performance of the SrV_4_O_9_-based electrode and the SrV_4_O_9_@rGO-based electrode. (**f**) Schematic illustration of the reaction mechanism of the SrV_4_O_9_@rGO-based electrode.

Figure 5a,b compare the CV curves of SrV_4_O_9_-based and SrV_4_O_9_@rGO-based electrodes for the first three cycles between 0.01 and 3.0 V at a scan rate of 0.1 mV s^−1^. All CV curves exhibited two reduction peaks and one oxidation peak. Two reduction peaks appeared at ~0.75 V and ~0.45 V, representing the penetration of sodium ions into SrV_4_O_9_. A weak and broad oxidation peak at around 1.48 V might correspond to the oxidation reaction of V. And the CV curves of the second and third cycles showed a high degree of overlap, indicating good reversibility of the electrochemical reactions. It is worth noting that the peak current densities of the oxidation–reduction peaks of the SrV_4_O_9_@rGO-based electrode (Figure 5b) were higher than those of the SrV_4_O_9_-based electrode (Figure 5a), indicating the improved electron/ion transport kinetics of the SrV_4_O_9_@rGO composite facilitated by rGO and more dispersed SrV_4_O_9_. The cycling performances of the SrV_4_O_9_-based and SrV_4_O_9_@rGO-based electrodes at 0.2 A g^−1^ are shown in Figure 5c. The SrV_4_O_9_@rGO composite showed a high initial reversible capacity of 284.4 mAh g^−1^ and maintained a stable capacity of 273.4 mAh g^−1^ after 200 cycles, with an ultra-high capacity retention of 96.1%. For the SrV_4_O_9_ electrode, the capacity after 200 cycles was only 142.5 mAh g^−1^. The excellent cycling performance can be attributed to the super aspect ratio of the porous microflower structure, which facilitated ion transfer. The introduction of rGO increased the specific surface area involved in the reaction, providing more reaction sites. The galvanostatic charge–discharge (GCD) curves of the SrV_4_O_9_@rGO electrode are shown in Figure 5d. These GCD curves show a high degree of overlap, indicating excellent cycling performance of the SrV_4_O_9_@rGO electrode. To characterize the positive effect of rGO on SrV_4_O_9_, an EIS test was performed. Figure S1 shows Nyquist plots for SrV_4_O_9_ and SrV_4_O_9_@rGO electrodes in the initial state and the SrV_4_O_9_@rGO electrode after 200 cycles. The SrV_4_O_9_@rGO electrode exhibited a smaller charge transfer resistance than the SrV_4_O_9_ electrode in the initial state, indicating that rGO improved the rate of Na^+^ transfer. A comparison between the initial and cycled electrodes revealed a significant decrease in impedance for the SrV_4_O_9_@rGO electrode after 200 cycles at 0.2 A g^−1^ due to the considerably enhanced ion and electron transfer in the electrode. The SEM image of the SrV_4_O_9_@rGO electrode after 200 cycles (Figure S2) revealed that the SrV_4_O_9_@rGO electrode had good structural stability after electrochemical testing. The SrV_4_O_9_@rGO electrode also demonstrated excellent rate performance (Figure 5e). At 0.1, 0.2, 0.5, 1.0, 2.0, 5.0, and 10.0 A g^−1^, the specific capacities of the SrV_4_O_9_@rGO electrode remained at 287.1, 268.5, 250.4, 228.7, 209.6, 168.2, and 120.4 mAh g^−1^, respectively, which were significantly higher than those of the SrV_4_O_9_ electrode (149.4, 119.0, 85.6, 69.2, 47.3, 23.1, and 7.7 mAh g^−1^, respectively). The cycling performance of the SrV_4_O_9_@rGO composite at a high rate (1 A g^−1^) was also tested, and results are shown in Figure S3. The SrV_4_O_9_@rGO composite delivered an initial reversible discharge capacity of 278.3 mAh g^−1^ at 1.0 A g^−1^. The capacity decreased slightly to 265.7 mAh g^−1^ after 310 cycles, demonstrating good cycling performance at a high rate. The improved electrochemical performance of the SrV_4_O_9_@rGO composite can be attributed to the large aspect ratio of the composite and improved electrical conductivity [43,44] (Figure 5f). 

## 3. Experimental Section

### 3.1. Materials

All reagents were directly used after purchase. Sr(OH)_2_∙8H_2_O, C_3_H_8_O_3_, V_2_O_5_, and H_2_O_2_ were purchased from Sigma-Aldrich (St. Louis, MO, USA). GO was provided by Nanjing XFNANO Materials Tech Co., Ltd. (Nanjing, China).

### 3.2. Synthesis of SrV_4_O_9_@rGO Composite and SrV_4_O_9_

The SrV_4_O_9_@rGO composite was prepared using the hydrothermal annealing method. First, 1 mmol of Sr(OH)_2_∙8H_2_O was dissolved in a mixed solution consisting of 10 mL of deionized water and 10 mL of C_3_H_8_O_3_. Next, 2 mmol (0.364 g) of V_2_O_5_ and 0.05 g of GO were added to a mixed solution consisting of 10 mL of deionized water and 5 mL of H_2_O_2_ (30%) and stirred for 1 h, and was then slowly added to the Sr(OH)_2_∙8H_2_O solution, followed by stirring for 2 h. The mixed solution was transferred to a hydrothermal reactor and heated in an oven at 200 °C for 48 h. After cooling to room temperature, the precipitate was collected after several washes with deionized water and ethanol. The collected material was then dried in a vacuum oven at 80 °C for 24 h. Finally, the collected material was heated to 450 °C at a heating rate of 5 °C min^−1^ in a tube furnace under argon and then held for 5 h to obtain SrV_4_O_9_@rGO. For comparison, SrV_4_O_9_ was prepared using the same procedures without the addition of GO.

### 3.3. Characterization

X-ray diffraction (XRD, Rigaku MiniFlexll, Rigaku Corporation, Tokyo, Japan) patterns were collected from 10° to 80° using Cu Kα radiation (λ = 1.5408 Å). X-ray photoelectron spectra (XPS, Thermo Scientific K-Alpha, Waltham, MA, USA) surveys and specific patterns were obtained with an Al Kα X-ray source. The structures of SrV4O9 and SrV_4_O_9_@rGO were further examined by Raman spectroscopy (Thermo Fisher DXR2xi). Morphologies of SrV_4_O_9_ and the SrV_4_O_9_@rGO composite were analyzed by scanning electron microscopy (SEM, JEOL JSM-7800F Field Emission, Manufacturer JEOL Ltd., Tokyo, Japan) with EDS mapping (JEOL JSM-7100F, Manufacturer JEOL Ltd., Tokyo, Japan) and transmission electron microscopy (TEM, JEOL JEM, 1011, Manufacturer JEOL Ltd., Tokyo, Japan). N_2_ adsorption–desorption isotherms were measured on an Autosorb-iQ instrument (Quantachrome, Anton Paar acquired Quantachrome Instruments, Inc., Boynton Beach, FL, USA).

### 3.4. Electrochemical Measurements

To prepare the working electrode, SrV_4_O_9_ or the SrV_4_O_9_@rGO composite (70 wt%) was combined with acetylene black (20 wt%) and polyvinylidene difluoride (10 wt%) in 1-methyl-2-pyrrolidone to form a uniform slurry. The slurry was then coated onto copper foil and dried at 80 °C for 12 h. The loading density of the active materials was about 1.2–1.5 mg cm^−2^. For electrochemical performance testing, a CR2025 coin cell was assembled with SrV_4_O_9_ or the SrV_4_O_9_@rGO composite as the working electrode, Na foil as the counter electrode, and glass fiber membrane (Whatman GF/A) as the separator. The electrolyte was 1 M NaClO_4_ dissolved in ethylene carbonate/dimethyl carbonate (EC/DMC, 1:1 by volume) with 5 wt% of fluorodimethylene carbonate (FEC) as an additive. Galvanostatic charge–discharge curves and cycling performance evaluations were performed on a LAND battery test system in the range of 0.01–3.0 V. Cyclic voltammetry (CV) tests were performed using the CHI660e electrochemical workstation.

## 4. Conclusions

In summary, an rGO-supported SrV_4_O_9_ composite was synthesized as an anode material for SIBs. The SrV_4_O_9_ had a regular micrometer-scale flower-like structure consisting of porous ultrathin nanosheets with a thickness of ~50 nm, which was coated with rGO to form a three-dimensional porous spherical structure. The porous nanostructure increased the contact area between SrV_4_O_9_@rGO and the electrolyte and effectively reduced the ion diffusion distance, thus improving ion transport efficiency. In addition, the porous nanostructure reduced SrV_4_O_9_ aggregation and provided sufficient buffering space for volume expansion during cycling processes, effectively improving cycling stability. Therefore, the SrV_4_O_9_@rGO composite showed a high initial reversible capacity of 284.4 mAh g^−1^ at 0.2 A g^−1^ and retained a stable capacity of 273.4 mAh g^−1^ after 200 cycles with an ultra-high capacity retention of 96.1%. At 0.1, 0.2, 0.5, 1.0, 2.0, 5.0, and 10.0 A g^−1^, the specific capacities of the SrV_4_O_9_@rGO electrode remained at 287.1, 268.5, 250.4, 228.7, 209.6, 168.2, and 120.4 mAh g^−1^, respectively, demonstrating excellent rate performance.

## Data Availability

Data are available upon request from the corresponding authors.

## Supplementary Materials

The following supporting information can be downloaded at https://www.mdpi.com/article/10.3390/molecules29112704/s1, Figure S1: Nyquist plots of the SrV4O9 and SrV4O9@rGO electrodes in the initial state and the SrV4O9@rGO electrode after 200 cycles; Figure S2: SEM image of the SrV4O9@rGO electrode after 200 cycles at 0.2 A g−1; Figure S3: Cycling performance of the SrV4O9@rGO composite after 200 cycles at 1 A g^−1^.

## References

[1] Gu S., Kong J., Fang B. (2024). Comprehensive recycling of spent lithium-ion batteries cathodes and anodes via targeted electrochemical redox process. Green Chem..

[2] Li J., Xie Q., Zhao Y., Zhao P., Zhang S., Huang W. (2024). Unveiling morphology evolution and performance enhancement of tin-doped Co_3_O_4_ porous nanoarrays anchored on stainless-steel mesh for advanced lithium-ion battery anodes. J. Energy Storage.

[3] Sun R., Qin Z., Li Z., Fan H., Lu S. (2020). Binary zinc-cobalt metal-organic framework derived mesoporous ZnCo_2_O_4_@NC polyhedron as a high-performance lithium-ion battery anode. Dalton Trans..

[4] Guo Y., Zhang X., Jin S., Xia Q., Chang Y., Wang L., Zhou A. (2023). Synthesis of Mo_2_C MXene with high electrochemical performance by alkali hydrothermal etching. J. Adv. Ceram..

[5] Fang B., Wang Y., Wang H. (2023). Does an LaCl_3_-based lithium superionic conductor work well for anode-free lithium metal batteries?. Matter.

[6] Li J., Lu Y., Quan K., Wu L., Feng X., Wang W. (2024). One-pot cocrystallization of mononuclear and 1D cobalt (II) complexes based on flexible triclopyr and 2, 2′-bipyridine coligands: Structural analyses, conformation comparison, non-covalent interactions and magnetic properties. J. Mol. Struct..

[7] Song M., Liu Y., Hong J., Wang X., Huang X. (2023). Boosting bidirectional conversion of polysulfide driven by the built-in electric field of MoS_2_/MoP Mott–Schottky heterostructures in lithium–sulfur batteries. J. Adv. Ceram..

[8] Zheng S., Mo L., Chen K., Chen A.-L., Zhang X., Fan X., Lai F., Wei Q., Miao Y.-E., Liu T. (2022). Precise Control of Li^+^ Directed Transport via Electronegative Polymer Brushes on Polyolefin Separators for Dendrite-Free Lithium Deposition. Adv. Funct. Mater..

[9] Li Z., Sun R., Qin Z., Liu X., Wang C., Lu S., Zhang Y., Fan H. (2021). Coupling of ReS_2_ nanosheet arrays with hollow NiCoS_4_ nanocubes enables ultrafast Na^+^ diffusion kinetics and super Na^+^ storage of a NiCoS_4_@ReS_2_ heterostructure. Mater. Chem. Front..

[10] Zhu J., He Q., Liu Y., Key J., Nie S., Wu M., Shen P.K. (2019). Three-dimensional, hetero-structured, Cu_3_P@C nanosheets with excellent cycling stability as Na-ion battery anode material. J. Mater. Chem. A.

[11] Zhu J., Wei P., Zeng Q., Wang G., Wu K., Ma S., Shen P.K., Wu X.-L. (2020). MnS@N,S Co-Doped Carbon Core/Shell Nanocubes: Sulfur-Bridged Bonds Enhanced Na-Storage Properties Revealed by In Situ Raman Spectroscopy and Transmission Electron Microscopy. Small.

[12] Wang Y., Xiao F., Chen X., Xiong P., Lin C., Wang H.-E., Wei M., Qian Q., Chen Q., Zeng L. (2023). Extraordinarily stable and wide-temperature range sodium/potassium-ion batteries based on 1D SnSe_2_-SePAN composite nanofibers. InfoMat.

[13] Zhou Y., Yin L., Xiang S., Yu S., Johnson H.M., Wang S., Yin J., Zhao J., Luo Y., Chu P.K. (2024). Unleashing the Potential of MXene-Based Flexible Materials for High-Performance Energy Storage Devices. Adv. Sci..

[14] Zhou Y.-L., Cheng W.-N., Bai Y.-Z., Hou C., Li K., Huang Y.-A. (2023). Rise of flexible high-temperature electronics. Rare Met..

[15] Lin H., Lin C., Xiao F., He L., Xiong P., Luo Y., Hu X., Qian Q., Chen Q., Wen Z. (2024). High-Performance Wide-pH Zn-Based Batteries via Electrode Interface Regulation with Valine Additive. Adv. Funct. Mater..

[16] Jia Y., Chen S., Shao X., Chen J., Fang D.-L., Li S., Mao A., Li C. (2023). Synergetic effect of lattice distortion and oxygen vacancies on high-rate lithium-ion storage in high-entropy perovskite oxides. J. Adv. Ceram..

[17] Zhou Y., Wang S., Yin J., Wang J., Manshaii F., Xiao X., Zhang T., Bao H., Jiang S., Chen J. (2024). Flexible Metasurfaces for Multifunctional Interfaces. ACS Nano.

[18] Lin C., He L., Xiong P., Lin H., Lai W., Yang X., Xiao F., Sun X.-L., Qian Q., Liu S. (2023). Adaptive Ionization-Induced Tunable Electric Double Layer for Practical Zn Metal Batteries over Wide pH and Temperature Ranges. ACS Nano.

[19] Li J., Liu M., You X., Wang J., Feng X. (2024). One-pot cocrystallization of 1D linear and zigzag cobalt (II) polymers assembled by triclopyr and 4, 4′-bipyridine: Structural comparison, conformational analysis, non-covalent interactions as well as the magnetic property of the latter. Polyhedron.

[20] Chen Y., Li F., Guo Z., Song Z., Lin Y., Lin W., Zheng L., Huang Z., Hong Z., Titirici M.-M. (2023). Sustainable and scalable fabrication of high-performance hard carbon anode for Na-ion battery. J. Power Sources.

[21] Zhao D., Zhang Z., Ren J., Xu Y., Xu X., Zhou J., Gao F., Tang H., Liu S., Wang Z. (2023). Fe_2_VO_4_ nanoparticles on rGO as anode material for high-rate and durable lithium and sodium ion batteries. Chem. Eng. J..

[22] Wang C., Wang Z., Zhao D., Ren J., Liu S., Tang H., Xu P., Gao F., Yue X., Yang H. (2021). Core–Shell Co_2_VO_4_/Carbon Composite Anode for Highly Stable and Fast-Charging Sodium-Ion Batteries. ACS Appl. Mater. Interfaces.

[23] Yang S., Zhang Y., Du Y., Wang Z., Song B., Wang X. (2023). SrV_4_O_9_ microflowers as high performance cathode for aqueous zinc-ion battery. Mater. Lett..

[24] Xia P., Li S., Yuan L., Jing S., Peng X., Lu S., Zhang Y., Fan H. (2024). Encapsulating CoRu alloy nanocrystals into nitrogen-doped carbon nanotubes to synergistically modify lithium-sulfur batteries separator. J. Membr. Sci..

[25] Wu S., Yang W., Liu Z., Li Y., Fan H., Zhang Y., Zeng L. (2024). Organic polymer coating induced multiple heteroatom-doped carbon framework confined Co_1-x_S@NPSC core-shell hexapod for advanced sodium/potassium ion batteries. J. Colloid Interface Sci..

[26] Gao F., Yue X.-A., Xu X.-Y., Xu P., Zhang F., Fan H.-S., Wang Z.-L., Wu Y.-T., Liu X., Zhang Y. (2023). A N/Co co-doped three-dimensional porous carbon as cathode host for advanced lithium–selenium batteries. Rare Met..

[27] Xu F., Li S., Jing S., Peng X., Yuan L., Lu S., Zhang Y., Fan H. (2024). Cobalt-vanadium sulfide yolk-shell nanocages from surface etching and ion-exchange of ZIF-67 for ultra-high rate-capability sodium ion battery. J. Colloid Interface Sci..

[28] Xu Y., Yu S., Yin Y., Bi L. (2022). Taking advantage of Li-evaporation in LiCoO_2_ as cathode for proton-conducting solid oxide fuel cells. J. Adv. Ceram..

[29] Qu Y.-P., Zhou Y.-L., Luo Y., Liu Y., Ding J.-F., Chen Y.-L., Gong X., Yang J.-L., Peng Q., Qi X.-S. (2024). Universal paradigm of ternary metacomposites with tunable epsilon-negative and epsilon-near-zero response for perfect electromagnetic shielding. Rare Met..

[30] Chen X., Zhou W., Wang F., Wu H., Zhong S., Li B. (2024). Meliorative dielectric properties in core@double-shell structured Al@Al_2_O_3_@PDA/PVDF nanocomposites via decoupling the intra-particle polarization and inter-particle polarization. Mater. Today Energy.

[31] Sun G., Yang D., Zhang Z., Wang Y., Lu W., Feng M. (2023). Oxygen vacancy-rich MoO_3_ nanorods as photocatalysts for photo-assisted Li–O_2_ batteries. J. Adv. Ceram..

[32] Wang F., Zhou W., He Y., Lv Y., Wang Y., Wang Z. (2024). Synergetic improvement of dielectric properties and thermal conductivity in Zn@ZnO/carbon fiber reinforced silicone rubber dielectric elastomers. Compos. Part A Appl. Sci. Manuf..

[33] Chen J., Yang Y., Yu S., Zhang Y., Hou J., Yu N., Fang B. (2023). MOF-Derived Nitrogen-Doped Porous Carbon Polyhedrons/Carbon Nanotubes Nanocomposite for High-Performance Lithium–Sulfur Batteries. Nanomaterials.

[34] Liao G., He Y., Wang H., Fang B., Tsubaki N., Li C. (2023). Carbon neutrality enabled by structure-tailored zeolite-based nanomaterials. Device.

[35] Fang B., Daniel L., Bonakdarpour A., Govindarajan R., Sharman J., Wilkinson D.P. (2021). Dense Pt Nanowire Electrocatal. Improv. Fuel Cell Perform. Using A Graph. Carbon Nitride-Decor. Hierarchical Nanocarbon Support. Small.

[36] Zhang Y., Zhou W., Peng W., Yao T., Zhang Y., Wang B., Cai H., Li B. (2024). Core@Double–Shell Engineering of Zn Particles toward Elevated Dielectric Properties: Multiple Polarization Mechanisms in Zn@Znch@PS/PVDF Composites. Macromol. Rapid Commun..

[37] Fang B., Kim J.H., Kim M.-S., Yu J.-S. (2013). Hierarchical Nanostructured Carbons with Meso–Macroporosity: Design, Characterization, and Applications. Acc. Chem. Res..

[38] Zhang F., Tang N., Jiang Q., Qi K., Zhu X., Luo Z., Kong X., Zang D., Liu H., Fang B. (2024). Progress in polyacrylate-based electrically conductive adhesives: Featured properties, preparation, applications, and perspectives. Polym. Compos..

[39] Zhang Z., Zhou J., Zhou X., Wang C., Pan Z., Xu X., Liu X., Wang Z., Wu Y., Jiang S. (2024). Graphene oxide-supported MnV_2_O_6_ nanoribbons with enhanced electrochemical performance for sodium-ion batteries. J. Power Sources.

[40] Zhang Y., Zhang Z., Yu S., Johnson H.M., Zhao D.-C., Tan S.-C., Pan Z.-D., Wang Z.-L., Wu Y.-T., Liu X. (2023). Three-dimensional nanostructured Co_2_VO_4_-decorated carbon nanotubes for sodium-ion battery anode materials. Rare Met..

[41] Jiang X., Li X., Kong Y., Deng C., Li X., Hu Q., Yang H., He C. (2023). A hierarchically structured tin-cobalt composite with an enhanced electronic effect for high-performance CO_2_ electroreduction in a wide potential range. J. Energy Chem..

[42] Li J., Liu X., Zhao H., Yang X., Xiao S., Liu N., Zhao N., Cao Y., Yu X., Li X. (2024). Dual-Phase engineering of Ni_3_S_2_/NiCo-MOF nanocomposites for enhanced ion storage and electron migration. Chem. Eng. J..

[43] Ding S., An J., Gao Y., Ding D., Lu X., Zhao L. (2023). Electrochemical performance of all-solid-state asymmetric supercapacitors based on Cu/Ni-Co (OH)_2_/Co_4_S_3_ self-supported electrodes. Chem. Eng. J..

[44] Senokos E., Anthony D.B., Rubio N., Ribadeneyra M.C., Greenhalgh E.S., Shaffer M.S. (2023). Robust single-walled carbon nanotube-infiltrated carbon fiber electrodes for structural supercapacitors: From reductive dissolution to high performance devices. Adv. Funct. Mater..

