# Supplementary material for: Reduced Graphene Oxide-Supported SrV4O9 Microflowers with Enhanced Electrochemical Performance for Sodium-Ion Batteries
Source: Molecules. 2024 Jun 6;29(11):2704. doi: 10.3390/molecules29112704 (PMC11173632; doi:10.3390/molecules29112704)
Supplement: Supplementary file 1 [file molecules-29-02704-s001.zip › molecules-3031061-supplementary.pdf]

Supporting Information

Reduced Graphene Oxide-Supported SrV_4O_9 Microflowers with Enhanced Electrochemical Performance for Sodium-Ion Batteries

Guangming Li [1,2], Yifan Li [2], Yi Zhang [2], Shuguo Lei [2], Jiwei Hou [2,*], Huiling Lu [3] and Baizeng Fang [4,*]

[1]*CNG Wind Energy Co., Ltd., Beijing 100160, PR China*

[2]*School of Physical and Mathematical Science, Nanjing Tech University, Nanjing 211816, Jiangsu, PR China*

[3]*School of Chemistry and Chemical Engineering, Jiangsu University, Zhenjiang 212013, Jiangsu, PR China*

[4]*School of Chemical Engineering and Energy Technology, Dongguan University of Technology, Dongguan 523808, Guangdong, PR China*

***Corresponding authors**:

jwhou@njtech.edu.cn (J. Hou)

baizengfang@163.com (B. Fang)

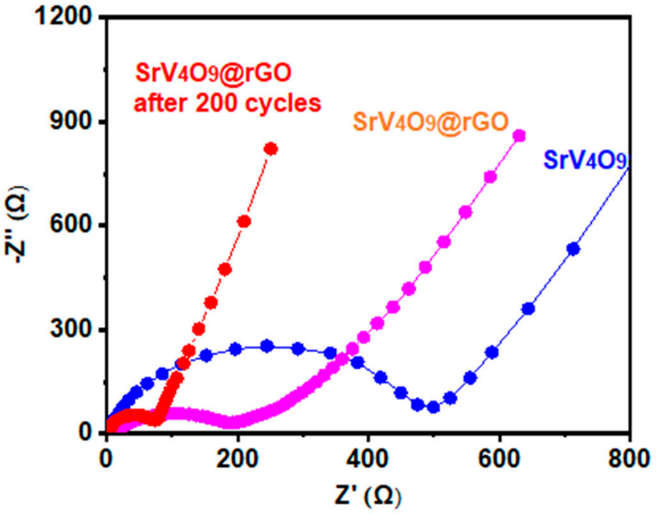

Figure S1. Nyquist plots of the SrV_4O_9 and SrV_4O_9@rGO electrodes in the initial state and the SrV_4O_9@rGO electrode after 200 cycles.

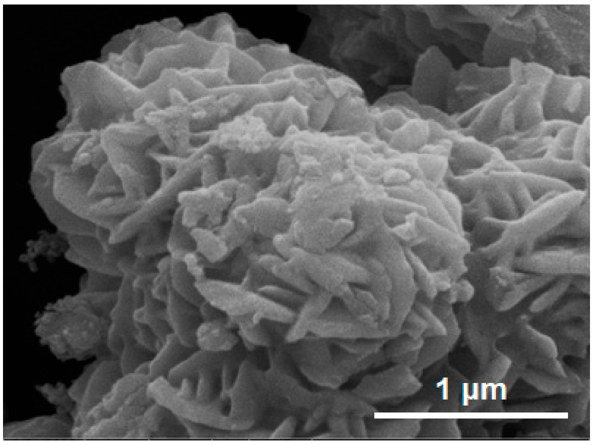

Figure S2. SEM image of the SrV_4O_9@rGO electrode after 200 cycles at 0.2 A g^{-1}.

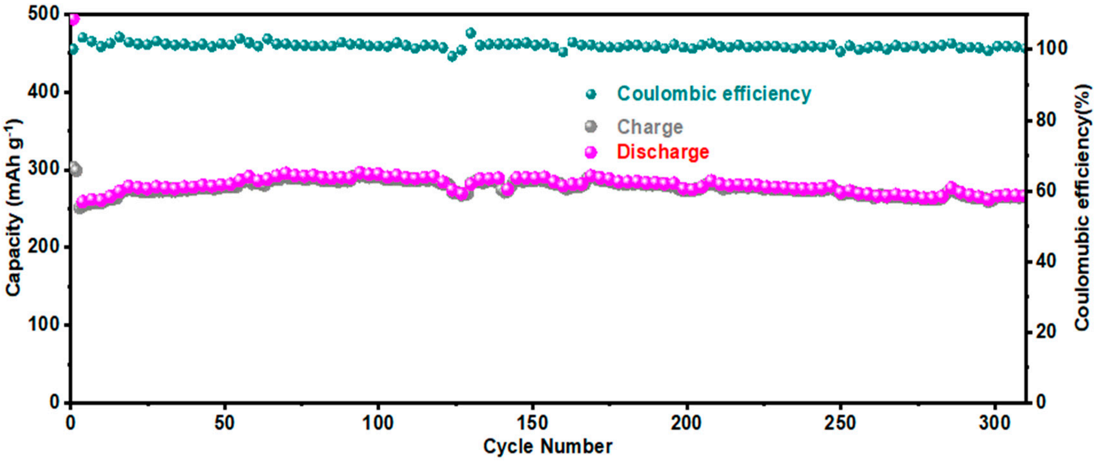

Figure S3. Cycling performance of the SrV_4O_9@rGO composite at 1.0 A g^{-1}.
